# Genome sequencing identifies “Limestone Canyon virus” as Montaño virus (*Hantaviridae: Orthohantavirus montanoense*) circulating in brush deermice in New Mexico

**DOI:** 10.1038/s44298-024-00016-6

**Published:** 2024-04-04

**Authors:** Samuel M. Goodfellow, Robert A. Nofchissey, Valerie J. Morley, Kathryn E. Coan, Kurt C. Schwalm, Joseph A. Cook, Jonathan L. Dunnum, Diane Hanfelt-Goade, Darrell L. Dinwiddie, Daryl B. Domman, Jerry W. Dragoo, Jens H. Kuhn, Steven B. Bradfute

**Affiliations:** 1 Center for Global Health, Department of Internal Medicine, University of New Mexico Health Sciences Center, Albuquerque, NM USA; 2 Museum of Southwestern Biology, Biology Department, University of New Mexico, Albuquerque, NM USA; 3 Department of Pediatrics, University of New Mexico Health Sciences Center, Albuquerque, NM USA; 4 Department of Pathology, University of New Mexico Health Sciences Center, Albuquerque, NM USA; 5 Integrated Research Facility at Fort Detrick, National Institute of Allergy and Infectious Diseases, National Institutes of Health, Fort Detrick, Frederick, MD USA; 6 Present Address: Creighton University School of Medicine, Phoenix, AZ USA; 7 Present Address: Te Whatu Ora Health New Zealand, Hauora a Toi Bay of Plenty, Tauranga, New Zealand; 8 Present Address: ABQ BioPark, Albuquerque, NM USA

**Keywords:** Viral evolution, Viral reservoirs, Virus structures

## Abstract

Orthohantaviruses infect distinct eulipotyphlan and rodent reservoirs throughout the world; some rodent orthohantaviruses can cause disease in humans. In the United States, a primary rodent reservoir for the human-pathogenic Sin Nombre virus (SNV) is the western deermouse (*Peromyscus sonoriensis*; formerly included in *Peromyscus maniculatus*). Deermice (rodents of genus *Peromyscus*) carry presumably distinct orthohantaviruses but, although deermice of ten species have been recorded in New Mexico, only SNV has been reported in rodents from that state. Using a set of pan-orthohantavirus primers, we discovered a non-SNV orthohantavirus in a brush deermouse (*P. boylii*), trapped in central New Mexico in 2019. Sequencing enabled the generation of a consensus coding-complete genome sequence, revealing similarity to the known partial sequences of the unclassified “Limestone Canyon virus (LSCV)” in GenBank and aligning with the information in an unpublished study of wild-caught brush deermice trapped in southwestern New Mexico in 2006. Phylogenetic analysis of these combined data revealed geospatial clades and overall identity of “LSCV”, uncovering its association with the classified Montaño virus (MTNV), which is known to infect Aztec and Orizaba deermice in central Mexico. Our work emphasizes the importance of determining coding-complete viral genome sequences as a framework for rigorous virus classification as the basis for epidemiological studies.

## Introduction

*Hantaviridae* is a bunyaviral family of trisegmented, enveloped, negative-sense RNA viruses that globally infect a wide range of hosts, including fish, reptiles, and mammals. Mammalian hantavirids are classified across four genera in the subfamily *Mammantavirinae*. Mammantavirins pathogenic for humans are rodent-borne viruses of genus *Orthohantavirus*^1^.

The first identified hantavirid, the orthohantavirus Hantaan virus (HTNV), was described in 1976 in striped field mice (Murinae: *Apodemus agrarius* (Pallas, 1771)) and identified as an etiologic agent of human “epidemic hemorrhagic fever” in (South) Korea^2,3^. Later identified in Asia and Europe were at least another ten orthohantaviruses that can cause the same disease^4^, now designated hemorrhagic fever with renal syndrome (HFRS; International Classification of Diseases 11th Revision [ICD-11] Code 1D62.0)^5^. All of these viruses are hosted by one or several distinct rodents^4^. The first identified American hantavirid, the nonpathogenic orthohantavirus Prospect Hill virus (PHV), was found in meadow voles (Arvicolinae: *Microtus pennsylvanicus* (Ord, 1815)) in Maryland in 1982^6^. This finding was followed by the discovery of Sin Nombre virus (SNV) in western deermice (Neotominae: *Peromyscus sonoriensis* (J. A. Wagner, 1845); recently split from *P. maniculatus*^7^) in the Four Corners region of the United States in 1993; SNV was quickly identified as the etiologic agent of a novel, severe, and often fatal human disease^8,9^ now designated hantavirus pulmonary syndrome (HPS; ICD-11 Code 1D62.1)^5^. Subsequently, ≈25 orthohantaviruses endemic to the Americas have been affiliated with HPS^4^, most notably Andes virus of long-tailed colilargos (Sigmodontinae: *Oligoryzomys longicaudatus* (Bennett, 1832)) in South America^10,11^. Currently, genus *Orthohantavirus* includes 38 species for 60 classified viruses^12^; more than 70 putative orthohantaviruses remain to be classified^13^.

Originally, distinct orthohantaviruses were hypothesized to be carried by and to co-evolve with distinct rodent hosts, but this school of thought has eroded considerably over recent years^1,14–19^. For instance, SNV may be maintained by rodents of multiple species, including least chipmunks (Sciuridae: *Neotamias minimus* (Bachman, 1839)), brush deermice (*Peromyscus boylii* (Baird, 1855)), and house mice (Murinae: *Mus musculus* Linnaeus, 1758) in addition to western deermice^20,21^. Seroprevalence studies have found anti-SNV antibodies in Piñon deermice (*Peromyscus truei* (Shufeldt, 1885)) and cactus deermice (*Peromyscus eremicus* (Baird, 1858))^22,23^. In addition, deermice of multiple species can be experimentally infected with SNV, with white-footed deermice (*Peromyscus leucopus* (Rafinesque, 1818)) developing higher viral and antibody titers than western deermice^24^. These findings indicate that incompletely characterized orthohantaviruses, which were given distinct names solely based on their “unique” rodent association and hence geographic distribution, may actually be known (and classified) orthohantaviruses. Intensive temporal and spatial surveillance efforts across diverse host communities, especially in areas of sympatry where multiple populations interact and may generate reassortant viruses, are needed for increased understanding of orthohantavirus ecology, evolution, and zoonotic potential^25,26^.

In the southwest region of the United States, including New Mexico, multiple *Peromyscus* species are syntopic, providing excellent opportunities to discover novel orthohantaviruses and study their ecology and evolution^27–29^. Coding-complete sequencing of orthohantavirus genomes assists with surveillance efforts^30–32^ and enables rigorous virus identification, taxonomic assignment^33^, and investigations of evolutionary processes (e.g., reassortment, phylodynamics). Using a pan-orthohantavirus real-time reverse transcription polymerase chain reaction (RT-qPCR) assay^30^ we surveyed deermouse populations throughout New Mexico and found a brush deermouse with a non-SNV orthohantavirus infection. Initial partial sequencing indicated the virus to be the unclassified “Limestone Canyon virus (LSCV)”, which was initially described in 2001 in brush deermice in central Arizona via partial sequencing of the small (S) and medium (M) genomic segments^34^. Generation of coding-complete “LSCV” S, M, and large (L) segment sequences identified the virus as Montaño virus (MTNV; *Orthohantavirus montanoense*), previously only known to infect Orizaba deermice (*Peromyscus beatae* (Thomas, 1903)) and Aztec deermice (*Peromyscus aztecus* (Saussure, 1860)) in central Mexico^35,36^. Furthermore, we found MTNV prevalence in multiple locations across New Mexico, revealing geospatially distinct subclades.

## Results

### Identification of an orthohantavirus in a wild-caught brush deermouse (*P. boylii*) in New Mexico

**Fig. 1:**
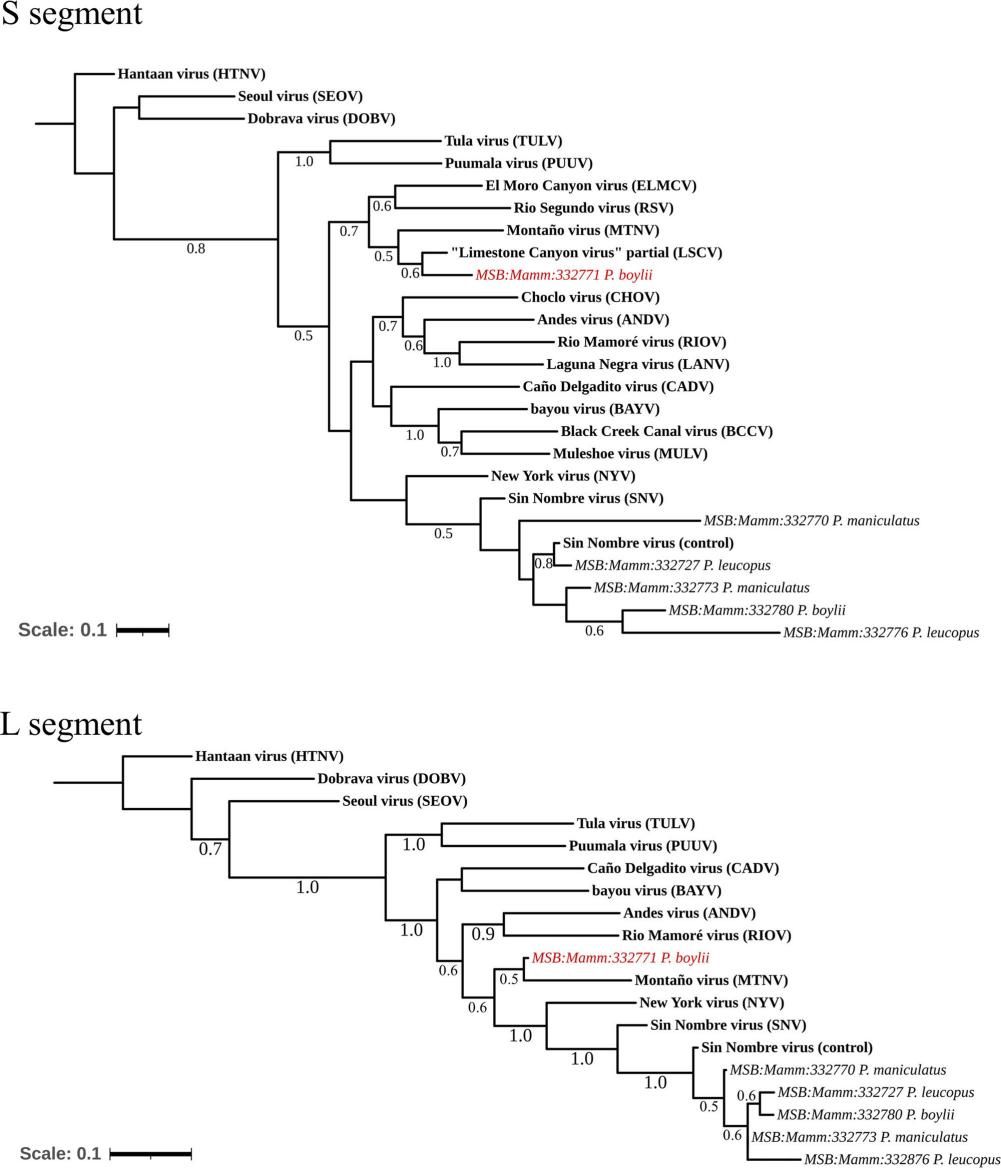
Phylogenetic tree of (S) and (L) fragment sequences. A maximum-likelihood tree using a General Time Reversible model was generated based on the partial nucleotide sequences of S segment (top) and L segment (bottom), using PanHS8 and Klempa primers, respectively. There are 1,000 bootstraps supported. *MSB:Mamm:332771* (red) indicates potential “LSCV” fragment sequence. The tree was rooted to the Hantaan virus (HTNV) reference sequence. Additional orthohantavirus reference sequences were obtained through NCBI. Sin Nombre virus (control) refers to SNV-infected Vero E6 supernatant used as a positive control. The scale bar represents the number of nucleotide substitutions per site.

In a previous study, we screened lung tissue of 100 wild-caught rodents captured in New Mexico to validate a novel pan-orthohantavirus RT-qPCR detection assay^30^. Using these primers to sequence amplified fragments of the S segment in the lungs of naturally infected wild-caught animals, we recovered a test-positive brush deermouse (MSB:Mamm:332771), trapped near a campground in Red Canyon, within the Manzano Mountains of central New Mexico (Table 1). The amplified 180-nucleotide (nt)-long sequence was 91.72% similar to the matching sequence of the 1,209-nt-long partial S segment sequence of “LSCV” in the National Center for Biotechnology Information (NCBI) GenBank database (#AF307322)^34^. Using a nested PCR assay against a hantavirid-conserved region of the L segment for hantavirids, we amplified and sequenced a 363-nt-long fragment with highest identity (79.19%) to MTNV (#AB620102), which has thus far only been described in samples from central Mexico^35,36^ (Fig. 1).

**Table 1:** “LSCV”-positive brush deermice (*P. boylii*) collected in two counties of New Mexico, USA

MSB Number	Date collected	County/Site	Sex	Total length (mm)	Tail length (mm)	Hind foot length (mm)	Ear length (mm)	Weight (g)	Reproduction status
MSB:Mamm:332771	09-28-2019	Torrance	Male	[155]	[64]	22	18	19	Non-scrotal
MSB:Mamm:264887	07-23-2006	Grant (Site 1)	Male	196	100	21	21	17	Non-scrotal
MSB:Mamm:264891	07-23-2006	Grant (Site 1)	Male	207	110	22	23	25	Scrotal
MSB:Mamm:264893	07-23-2006	Grant (Site 1)	Male	174	80	21	23	24.5	Non-scrotal
MSB:Mamm:264923	07-29-2006	Grant (Site 2)	Male	211	118	23	21	25	Non-scrotal
MSB:Mamm:264924	07-29-2006	Grant (Site 2)	Female	202	109	21	22	24.5	Closed, no scars
MSB:Mamm:264927	07-29-2006	Grant (Site 2)	Male	199	97	21	22	25	Scrotal
MSB:Mamm:264934	08-20-2006	Grant (Site 2)	Male	207	104	22	22	28	Scrotal
MSB:Mamm:264937	08-20-2006	Grant (Site 3)	Male	203	110	22	21	25	Scrotal

Species affiliation was confirmed initially by morphology assessment followed by cytochrome b gene sequencing. MSB:Mamm:332771 brackets signify the total measurements and tail are short due to a tail bob. *MSB* Museum of Southwestern Biology.

Brush deermice were measured and collected in two counties at different time points.

High-throughput sequencing (HTS) strategies have been increasingly utilized for viral identification and surveillance in host tissues^50–52^. Only recently have they been applied to orthohantaviruses, but low viral titers in host tissues pose difficulty for accurate sequencing and identification^50^. Due to the lack of a coding-complete “LSCV” genome sequence (available sequences only partially covered S and M sequences^34,53,54^), we conducted whole-genome sequencing (WGS) to assemble a coding-complete genome of the virus from MSB:Mamm:332771. Of 14,907,288 reads, 44,667 were virus-related, with 38,325 (85.8%) associated with orthohantaviruses. Top hits were (in descending frequency): MTNV (9,514—24.8%), Khabarovsk virus (KHAV; 6761—17.6%), SNV (3989—10.4%), Caño Delgadito virus (CADV; 1,263—3.3%), Fǔsōng virus (FUSV; 1274—3.3%), Rockport virus (RKPV; 913—2.4%), and HTNV (921—2.4%) (Supplemental Fig. 1). Interestingly, MTNV was the top hit for reads associated with L sequence fragment data generated with RT-qPCR (Fig. 1).

We filtered our trimmed reads to only those that binned to *Hantaviridae*. Next, we performed *de novo* sequence assembly and generated 18 nodes. Then, we screened each node using NCBI Basic Local Alignment Search Tool (NCBI BLAST), with eight nodes generating hits for hantavirids and contigs matching the MSB:Mamm:332771 virus genome sequence. Of the eight contigs, four formed the S segment (nucleoprotein coding region), one formed the M segment (coding region for the Gn/Gc glycoproteins), and three formed the L segment (coding region for the large protein with its RNA-directed RNA polymerase). Using MTNV (#AB620100–AB620102) as a reference genome guide due to similarities, S and L segment sequences appeared mostly complete after aligning contigs. The M segment sequence only contained 1,352 nt but was anticipated to be no less than 3489 nt (Supplemental Table 1). We aligned our sequence with the previously reported “LSCV” M segment sequence (#AF307323)^34^ and measured 92.97% nucleotide identity. Using the reported M segment sequence (#AF307323) and MTNV L segment fragment sequence (#AB620102), we optimized and re-designed primer sets (Supplemental Table 2) and Sanger-sequenced the missing parts of the M and L segments to generate a coding-complete sequence.

We verified the sequence from the WGS using an amplicon-based PCR tiling approach that has been frequently used to sequence orthohantavirus genomes^21,31,32^. We generated 19 different primer sets that overlapped and spanned the coding regions of the S, M, and L segments (Supplemental Table 2) based on the sequence we derived using WGS. Using MSB:Mamm:332771 lung tissue cDNA, we tested each primer set and amplified the entire coding region of the virus genome (Supplemental Fig. 2). Amplified products were purified and subjected to Sanger sequencing. We aligned the sequence fragments with the WGS-generated genome sequence and found 99.7% sequence homology. Any nucleotide disagreement was manually viewed to ensure nucleotide accuracy. This additional validation step assured a consensus genome for MSB:Mamm:332771 and the accuracy of our viral WGS approach. The final sequences were deposited to NCBI GenBank under accession numbers #OR148902–4.

### Extension of partial orthohantavirus sequences in brush deermouse (*P. boylii*) specimens collected in southwestern New Mexico

In 2006, wild-caught rodents in Hidalgo and Grant counties in southwestern New Mexico were surveyed (Supplemental Table 3). Of the 88 deermice captured, brush deermice (*P. boylii*) accounted for ≈ 65% (57/88), along with ≈17% Piñon deermice (*P. truei*, 15/88), ≈16% cactus deermice (*P. eremicus*, 14/88), ≈1% western deermice (*P. sonoriensis*, 1/88), and ≈1% white-footed deermice (*P. leucopus*, 1/88). Serologically, 33 of the 88 mice had immunoglobulin G (IgG) antibodies that cross-reacted with SNV N antigen, whereas 16 of the 33 cross-reacted to Rio Mamoré virus (RIOMV) N antigen. Most positives were brush deermice (28 seropositive for SNV and 16 for RIOMV), whereas only four Piñon deermice and one cactus deermouse were seropositive for SNV.

**Fig. 2:**
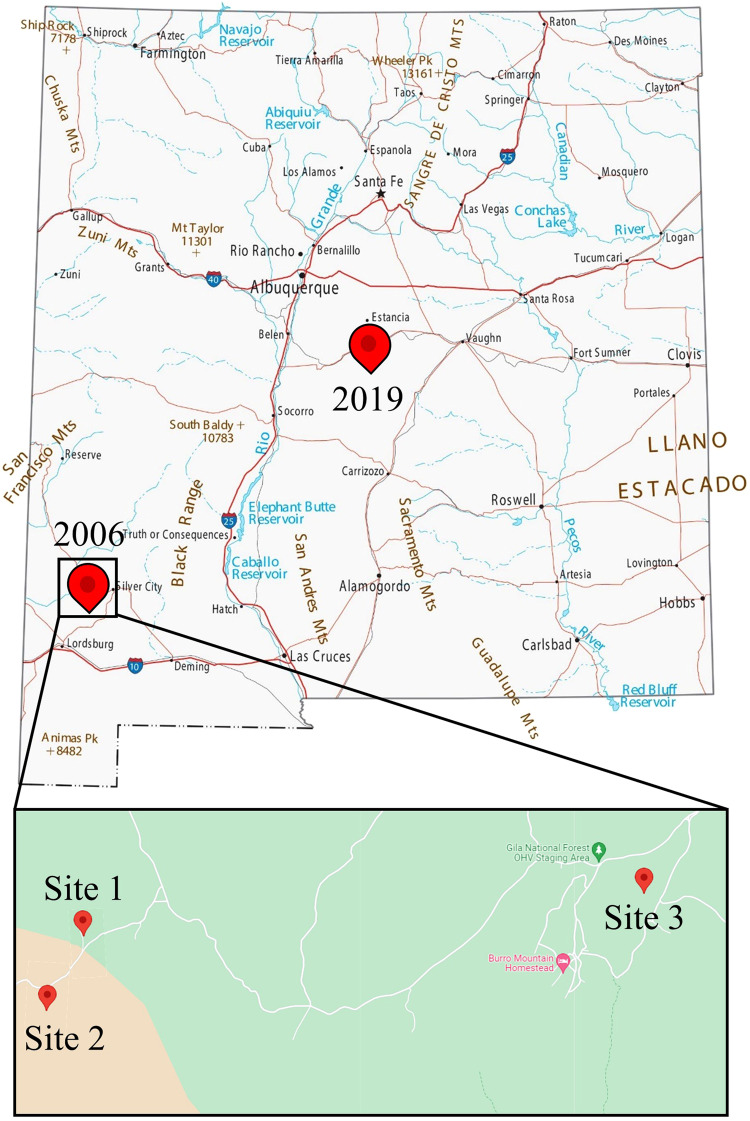
Map of locations of “LSCV”-positive rodents collected in New Mexico. Red markers indicate collection localities of specimens used for this study. Enlarged area shows multiple collection sites from 2006. Source: New Mexico map and Google maps.

As previously described^55^, levels of seropositivity did not necessarily correlate with detection of viral genetic material, fragments of which were only detected in eight specimens of *P. boylii* by custom designed primers in an RT-PCR assay targeting the S segment. These specimens were acquired from three sites designated Site 1, Site, 2, and Site 3. Site 1 and Site 2 are ≈0.6 km apart, with a road between them, whereas Site 3 is located ≈8 km into the forest region near Burro Peak in the Big Burro Mountains. A 2019 sample (MSB:Mamm:332771) was collected in central New Mexico, whereas the previous study was conducted in the southwestern region of the state, roughly 400 km apart (Fig. 2). Partial M segment sequences could be generated from seven of these eight specimens. The S and M segment sequences diverged by 5.4–6.4% and 5.0–6.5% from the previously deposited “LSCV” S (#AF307322) and M (#AF307323) segment sequences^34^ (Table 1, Fig. 1).

To extend analysis of this virus in the samples collected in 2006, we retrieved lung tissues for the eight specimens from the University of New Mexico (UNM) Museum of Southwestern Biology (MSB) Division of Genomic Resources (DGR) biorepository and performed RT-qPCR. We successfully expanded the S segment sequences from ≈1200–1600 nt and the M segment fragments from ≈800–900 to ≈3000 nt We also generated partial L segment sequences for all eight rodents.

### Geospatial clustering of orthohantavirus sequences in brush deermice (*P. boylii*)

**Fig. 3:**
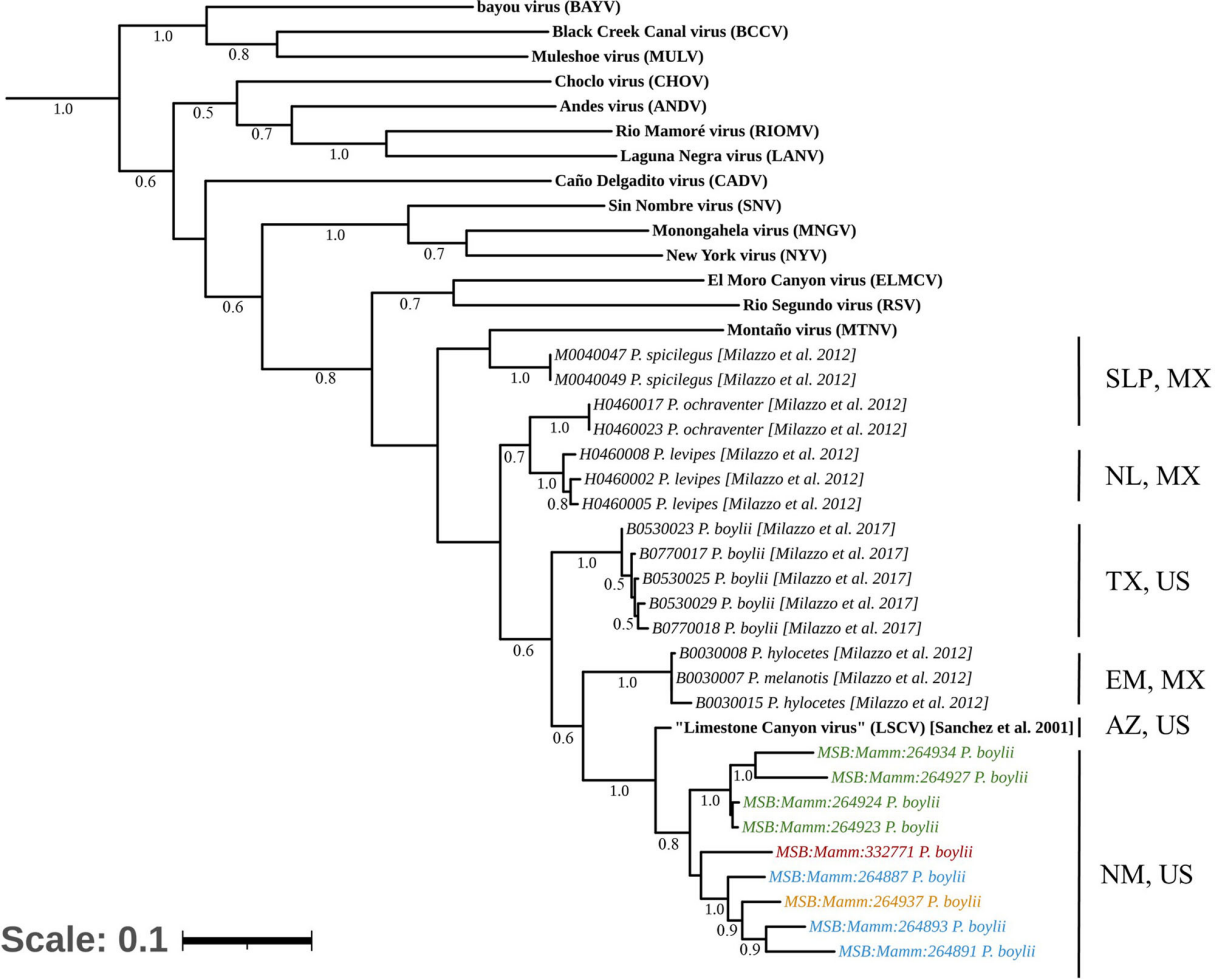
Phylogenetic tree of the “LSCV” S Segment. A maximum-likelihood tree was generated using a General Time Reversible model based on nucleotides of the S segment (nucleocapsid coding region) of multiple reported sequences of orthohantaviruses. There are 1,000 bootstraps supported. *MSB:Mamm:332771* (red) indicates reference genome generated. Samples from New Mexico formed a monophyletic clade composed of two subclades, delineating further geographic structure and containing samples from Site 1 (blue), Site 2 (green), and Site 3 (gold). Additional orthohantavirus reference sequences were obtained through NCBI. The scale bar represents the number of nucleotide substitutions per site. US United States, MX Mexico, TX Texas, SLP San Luis Potosí, NL Nuevo León, EM State of Mexico, AZ Arizona, NM New Mexico.

**Fig. 4:**
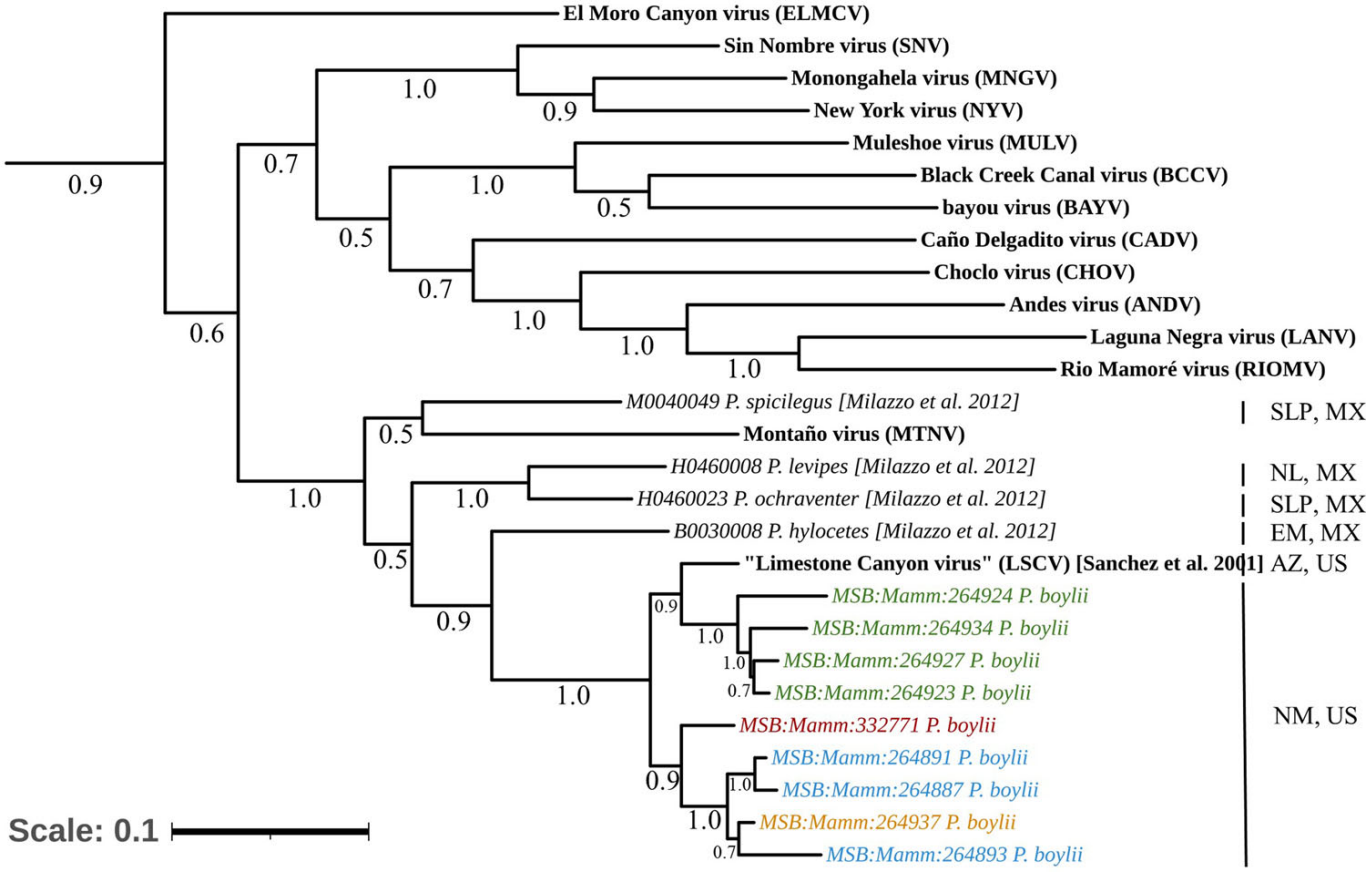
Phylogenetic tree of the “LSCV” M Segment. A maximum-likelihood tree was generated using a General Time Reversible model based on nucleotides of the M segment (glycoproteins Gn/Gc coding region) of multiple reported sequences of orthohantaviruses. There are 1,000 bootstraps supported. *MSB:Mamm:332771* (red) indicates reference genome generated. Samples from New Mexico formed a monophyletic clade composed of two subclades, delineating further geographic structure and containing samples from Site 1 (blue), Site 2 (green), and Site 3 (gold). Additional orthohantavirus reference sequences were obtained through NCBI. The scale bar represents the number of nucleotide substitutions per site. US United States, MX Mexico, TX Texas, SLP San Luis Potosí, NL Nuevo León, EM State of Mexico, AZ Arizona, NM New Mexico.

Because the two studies were conducted in different regions of New Mexico, we analyzed the genetic similarity of the generated sequences against the “LSCV” fragments published previously from Arizona^34^, ≈574–716 km from the New Mexico sites, along with other previously reported fragments in GenBank^53,54^. We performed phylogenetic analysis to compare individual segments against known reference sequences of diverse hantavirids. All S segment sequences grouped under “LSCV”. Interestingly, we observed that samples from New Mexico clustered together, apart from fragments reported from Texas and Mexico. However, Site 1 and Site 2 sequences from the 2006 study clustered separately. The initial “LSCV” fragment from Arizona was most similar to sequences from New Mexico than to those from Texas or Mexico (Fig. 3). The “LSCV” M segment sequences repeated this pattern; Site 1 and Site 3 clustered apart from Site 2 sequences. The 2019 MSB:Mamm:332771 sequence clustered with New Mexico sequences (Fig. 4).

**Fig. 5:**
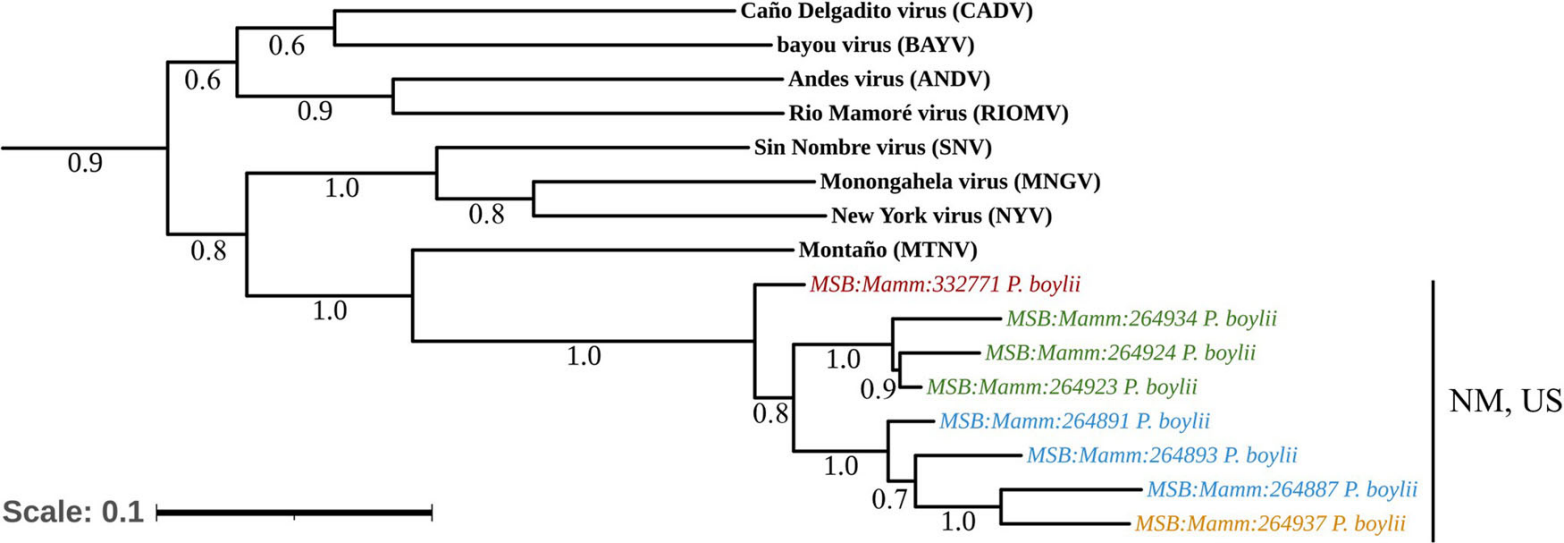
Phylogenetic tree of the “LSCV” L Segment. A maximum-likelihood tree was generated using a General Time Reversible model based on nucleotides of the L segment (RNA-dependent RNA polymerase) of multiple reported sequences of orthohantavirusess. There are 1,000 bootstraps supported. *MSB:Mamm:332771* (red) indicates reference genome generated. Samples from New Mexico formed a monophyletic clade composed of two subclades, delineating further geographic structure and containing samples from Site 1 (blue), Site 2 (green), and Site 3 (gold). Partial fragments were generated for MSB:Mamm:264927 so both were aligned and mapped to reference sequences and specimens. Additional orthohantavirus reference sequences were obtained through NCBI. The scale bar represents the number of nucleotide substitutions per site. US United States, MX Mexico, TX Texas, SLP San Luis Potosí, NL Nuevo León, EM State of Mexico, AZ Arizona, NM New Mexico.

Because there were no previously published sequences for the L segment of “LSCV”, we placed our sequences in a phylogenetic framework by alignment. Similar to the S and M segment sequences, the L segment sequences from New Mexico clustered together and the MSB:Mamm:332771 sequence diverged (Fig. 5).

A comparison of the total coding “LSCV” sequence spanning all three segments against other known orthohantaviruses revealed that the closest relative is MTNV. Using MSB:Mamm:332771, since it is the only “LSCV” sequence with complete coding regions, we analyzed the percent identity between virus genome sequences. Compared to MTNV sequences, we found 29.9, 22.1, and 6.5% nucleotide divergence and 6.5, 11.8, and 9.6% deduced amino-acid sequence divergence for the S, M, and L segment sequences, respectively (Supplemental Fig. 3).

Finally, we analyzed the deduced protein sequences against other known reference sequences. As expected, the “LSCV” sequence from MSB:Mamm:332771 grouped with other orthohantaviruses from the Americas and most closely with MTNV (Supplemental Fig. 4). According to the updated International Committee on Taxonomy of Viruses (ICTV) *Hantaviridae* Study Group guidelines, orthohantavirus species can only be established (or maintained) when at least one coding-complete exemplar virus genome (S + M + L segment) sequence is available and if this sequence sufficiently differs from others in DivErsity pArtitioning by hieRarchical Clustering (DEmARC) analysis^56^ (updated from Laenen, L. et al.^57^). DEmARC analysis of the now available coding-complete “LSCV” genome sequence identified it as MTNV^56^. Although “LSCV” was described prior to the description of MTNV, the MTNV coding-complete genome was deposited in 2012, giving MTNV priority^34–36^. Consequently, the terms “Limestone Canyon virus” and “LSCV” should be considered junior synonyms of “Montaño virus” and “MTNV”, respectively, and hence ought to be abandoned.

## Discussion

Early studies suggested that orthohantaviruses and their specific rodent hosts co-evolved, with only occasional species-jumping events^19,34,58^. Instead, numerous recent studies indicate that such events may occur frequently and that rodents of multiple species can become infected by the same virus^17,22–24,59,60^. Indeed, we and others have shown that rodents from multiple species and genera in a single community can be infected with SNV, diminishing the concept of mammalian species being a barrier for orthohantavirus infection of multiple host reservoirs^20,21^.

For this study, we determined the coding-complete genome sequence of “LSCV” and found a novel lineage thereof in New Mexico brush deermouse (*P. boylii*) populations. Using this sequence, we demonstrated “LSCV” to be synonymous with MTNV, which was previously found in deermice of multiple species, such as Aztec and Orizaba deermice in Mexico^35,36^. Because “LSCV” was previously shown to infect deermice of multiple species, MTNV can now be considered rather promiscuous and geographically broadly distributed^53,54^. The pathogenic potential of MTNV is unknown and requires investigation.

In New Mexico, the distributions of deermice of multiple species overlap, suggesting that there is high potential for multiple orthohantaviruses to co-occur in the same area and even in the same individual rodent^59,61^. Understanding geographic variation in hosts and associated orthohantaviruses provides a framework for interpreting evolutionary and ecological processes underlying these pathogens^17^. Additional efforts to survey brush deermouse populations as well as deermice in general will be necessary to establish the boundaries and distribution of populations to help assess the potential for intra- and inter-species genomic segment reassortment and hence the possibility for novel orthohantavirus emergence.

## Methods

### Ethics statement

All field procedures were performed following the animal care and use guidelines of the American Society of Mammalogists^37^. The protocol was approved by the UNM Institutional Animal Care and Use Committee, and specimens were collected under a New Mexico Department of Game and Fish permit to J.A.C. (authorization number 3300). Holistic museum specimens were prepared according to the best practices for emerging pathogen research and databased in a relational collection management system (https://arctosdb.org) to facilitate the linkage of host specimen data and derived pathogen data, which include the exact latitude and longitude for each trap location^38,39^.

### Trapping and sample collection

In late September 2019, 80 Sherman traps (38 × 3.59 × 239 cm; H.B Sherman Co., Tallahassee, Florida, USA) were placed near the upper Red Canyon campground for one night. Traps were baited with a peanut-butter-and-oat mixture, set out in the late evening, and collected early the next morning. Nine rodents were captured as follows: five western deermice (*P. sonoriensis*, 56%), three brush deermice (*P. boylii*, 33%), and one white-throated woodrat (Neotominae: *Neotoma albigula* (Hartley, 1894, 11%); overall trapping success was 11.3%. Collected tissues were snap-frozen on dry ice and included brown fat, spleen, heart, lungs, kidneys, liver, colon (with feces if present), urinary bladder (with urine if present), and serum from whole blood collected by cardiac puncture. All samples were stored at −80 °C until processing.

In 2006, samples were similarly collected in Granite Gap and multiple sites in the Big Burro Mountains in Hidalgo and Grant Counties, New Mexico, placed in liquid nitrogen in the field, and later stored at −80 °C. Tissues (liver, spleen, heart/kidney, and lung) were obtained from museum specimens for confirmation of animal identification and detection of viruses. Holistic voucher specimens were prepared and are archived in the UNM MSB DGR (Supplemental Table 3).

Lung tissue from samples collected in 2006 were retrieved from the MSB DGR biorepository for MSB:Mamm:264887, MSB:Mamm:264891, MSB:Mamm:264893, MSB:Mamm:264923, MSB:Mamm:264924, MSB:Mamm:264927, MSB:Mamm:264934, and MSB:Mamm:264937.

### Rodent identification

Standard measurements (total length, tail length, hind foot length [with claw], ear length [from notch], weight, reproductive data [sex, reproductive status, and testes for males, and, if pregnant, embryo crown–rump measurements]) and age were recorded. Species identifications were conducted through a combination of measurement data and morphological characters and confirmed through mitochondrial cytochrome b (CYB) gene sequence analysis. CYB was amplified using 15334L (5’-CTTCATTTTTGGTTTACAAGAC-3’) and L14724 (5’-TGATATGAAAAACCATCGTTG-3’)^40^. The PCR products were purified using the QIAquick purification PCR Kit (catalog number 28104; Qiagen, Germantown, Maryland, USA) and sent to Sequetech (Mountain View, California, USA) with the 15334L primer for sequencing. CYB gene sequences of the mitochondrial DNA were used to verify the species assignments of the mice captured in 2006. Approximately 10 mg of liver tissue was used to attain DNA via salt extraction^41^. PCR was performed using primers L14724 and H15915^42^. Cleaned PCR products were sequenced using BigDye Terminator Cycle Sequencing Ready Reaction mix v1.1 (catalog number 4337449, Applied Biosystems, Waltham, Massachusetts, USA) and the forward primer L14724. Sequence reactions were run on an ABI 3100 automated DNA sequencer in the Molecular Biology Facility in the UNM Biology Department. Cleaned sequences from these mice were analyzed by comparing them to GenBank sequences reported by Tiemann-Boege et al.^43^ in a Neighbor Joining analysis to confirm the identity of each mouse.

### Serology

Serology was performed on the 2006 samples using blood collected by cardiac puncture. The presence of IgG antibodies to SNV nucleoprotein (N) protein (SNV N) and RIOMV N was determined using a strip immunoblot assay (SIA) according to the protocol previously described in Yee et al.^44^. Briefly, each SIA strip was prepared using a model SB 10 mini slot blot apparatus and had an orientation band of Coomassie blue dye and control bands of 3+ IgG intensity, SNV N antigen, RIOMV N antigen, and a 1+ IgG intensity. Components were vacuum-blotted onto nitrocellulose membrane, allowed to dry, and cut into 2-mm strips. Blood samples were pre-blocked for 30 min in a milk buffer before adding the SIA strips and were incubated overnight on a rocker. Strips were washed three times before adding a goat anti-mouse IgG (H + L) alkaline phosphatase (AP) conjugated secondary antibody (KPL catalog number 475–1806) at a 1:1,000 dilution in phosphate milk buffer and allowing them to incubate with rocking at room temperature for 1 h. Then, strips were washed three times before adding the standard AP developing solution for 20 min to visualize bands. Strips were washed a final three times in ddH_2_O to stop the reaction. Bands were evaluated while wet and assigned scores using an intensity scale of 0–4.

### RNA extraction

RNA extraction for MSB:Mamm:332771, MSB:Mamm:264887, MSB:Mamm:264891, MSB:Mamm:264893, MSB:Mamm:264923, MSB:Mamm:264924, MSB:Mamm:264927, MSB:Mamm:264934, and MSB:Mamm:264937 was performed using the QIAmp Viral RNA Mini Kit (catalog number 52906, Qiagen) according to the manufacturer’s instructions with slight modifications. A total of 40 mg of frozen lung tissue was homogenized using a BeadBug 6 Microtube Homogenizer (Benchmark Scientific, Sayreville, New Jersey, USA) in a bead beater tube preloaded with 1.0 g of 1.0-mm-diameter zirconia beads (catalog number 1107911zx; BioSpec, Bartlesville, Oklahoma, USA), 1.0 g of 2.0-mm-diameter zirconia beads (catalog number 11079124zx; BioSpec), and 600 mL of AVL buffer. The tissue was beaten at 4,350 rpm for 30 s for one cycle. Homogenates were centrifuged, placed in a new 1.5-mL microcentrifuge tube, and re-centrifuged to remove any excess debris. RNA carrier was added to the clear lysate, and RNA isolation proceeded per the manufacturer’s instructions. RNA was extracted from lung tissue samples collected in 2006 using the standard protocols for the RNeasy kit (catalog number 74106; Qiagen).

### Primer design

PanHS8 was used to detect and provide partial sequence for the S segment of “LSCV” for MSB:Mamm:332771^30^.

Samples from 2006 were initially unsuccessfully tested using previously published primers^34,45^. Subsequently, primers were designed by submitting “LSCV” sequences to Primer3 (https://primer3.ut.ee/) for analyses^46^. Multiple primers were designed for both the S and M segments, but only two worked for the S segment (LSCS 114–5’-AGTGGACCCGGATGATGTTA-3’ and LSCS 1117−5’-TACGTCGGAGGTAGGATTGG-3’) and three for the M segment (LSCM 2077−5’-ATCCTTGGTCATTGGATGA-3’, LSCM 303−5’-GAATGGCCTCCCTTTCCTAC-3’, and LSCM 3312−5’-TGTGAACGAATGGGACAGAA-3’). Cleaned PCR products were sequenced with the amplification primers using the ABI PRISM BigDye Terminator Cycle Sequencing Ready Reaction (Applied Biosystems). Sequences were determined by running samples on an ABI 3100 automated DNA Sequencer (Applied Biosystems).

### RT-qPCR and nested PCR

RT-qPCR and nested PCR for MSB:Mamm:332771 were performed as previously described by Goodfellow, S. M. et al. and Klempa, B. et al., respectively^30,47^.

For MSB:Mamm:264887, MSB:Mamm:264891, MSB:Mamm:264893, MSB:Mamm:264923, MSB:Mamm:264924, MSB:Mamm:264927, MSB:Mamm:264934, and MSB:Mamm:264937, the presence of RNA was confirmed using absorbance spectrometry. RNA was converted into a cDNA library using the Ominscript Reverse Transcription kit (catalog number 205113; Qiagen). A poly(T) primer was used to construct cDNA. An attempt to amplify the M segment was only performed on the sample from the single mouse in which the S segment was amplified.

### WGS and *de novo* assembly

An Illumina sequencing library from MSB:Mamm:332771 RNA from lung tissue was created using the KAPA RNA HyperPrep Kit with RiboErase (HMR) following manufacturer’s recommended protocol (catalog number 8098131702;Roche, Indianapolis, Indiana, USA). The library was sequenced by TGen (https://tgen.org ;Phoenix, Arizona, USA) on a NovaSeq 6000 (Illumina ;San Diego, California, USA). Pair-end reads (2×150-nt) were generated per sample and transferred to personal database followed by trimming of adapters using TrimGalore v0.6.1 (https://www.bioinformatics.babraham.ac.uk/projects/trim_galore/). Running Kraken2 database, trimmed reads were visualized using KronaTools to generate Krona plots (Supplemental Fig. 1)^40,48^. After identification, all bunyaviral reads including sub-taxa were extracted. SPAdes Genome Assembler, specifically rnaviralSPAdes, was then run on extracted reads, generating 18 nodes that were put into BLAST to check for orthohantavirus hits^49^. Eight of the 18 nodes were positive and were aligned for each segment accordingly using CLUSTAL Omega. Gaps for M segment sequences were filled in by Sanger sequencing using primers generated from “LSCV” fragment (#AF307323) in NCBI GenBank.

### Phylogenetic analysis

Samples from both studies were aligned with CLUSTAL Omega (https://www.ebi.ac.uk/Tools/msa/clustalo/) and Jalview (https://www.jalview.org/). Molecular Evolutionary Genetics Analysis (MEGA-X - https://www.megasoftware.net/) was used to construct a maximum-likelihood tree with 1,000 bootstraps in a General Time Reversible model based on nucleotides shown to provide a uniform substitution matrix. Phylogenetic trees were generated using Interactive Tree of Life (iTOL - https://itol.embl.de/). CLUSTAL Omega was also used to produce percent identity matrices. Sequences used in the analysis are listed in Supplemental Table 4. ExPASy was used to convert nucleotides into amino acids to view variants.

## Supplementary Information

Supplemental Figures and Tables

## Data Availability

Sequences were deposited in GenBank. MSB:Mamm:264887 (OR552617–619), MSB:Mamm:264891 (OR552620–622), MSB:Mamm:264893 (OR552623–625), MSB:Mamm:264923 (OR552604–606), MSB:Mamm:264924 (OR552611–613), MSB:Mamm:264927 (OR552607–610), MSB:Mamm:264934 (OR552601–603), MSB:Mamm:264937 (OR552614–616), and MSB:Mamm:332771 (OR148902–OR148904). All museum metadata is available via Arctos (https://arctosdb.org).
