# Supplementary material for: Genome sequencing identifies “Limestone Canyon virus” as Montaño virus (Hantaviridae: Orthohantavirus montanoense) circulating in brush deermice in New Mexico
Source: Npj Viruses. 2024 Apr 4;2:11. doi: 10.1038/s44298-024-00016-6 (PMC11721154; doi:10.1038/s44298-024-00016-6)
Supplement: Supplementary file 1 — Supplemental Figures and Tables [file 44298_2024_16_MOESM1_ESM.pdf]

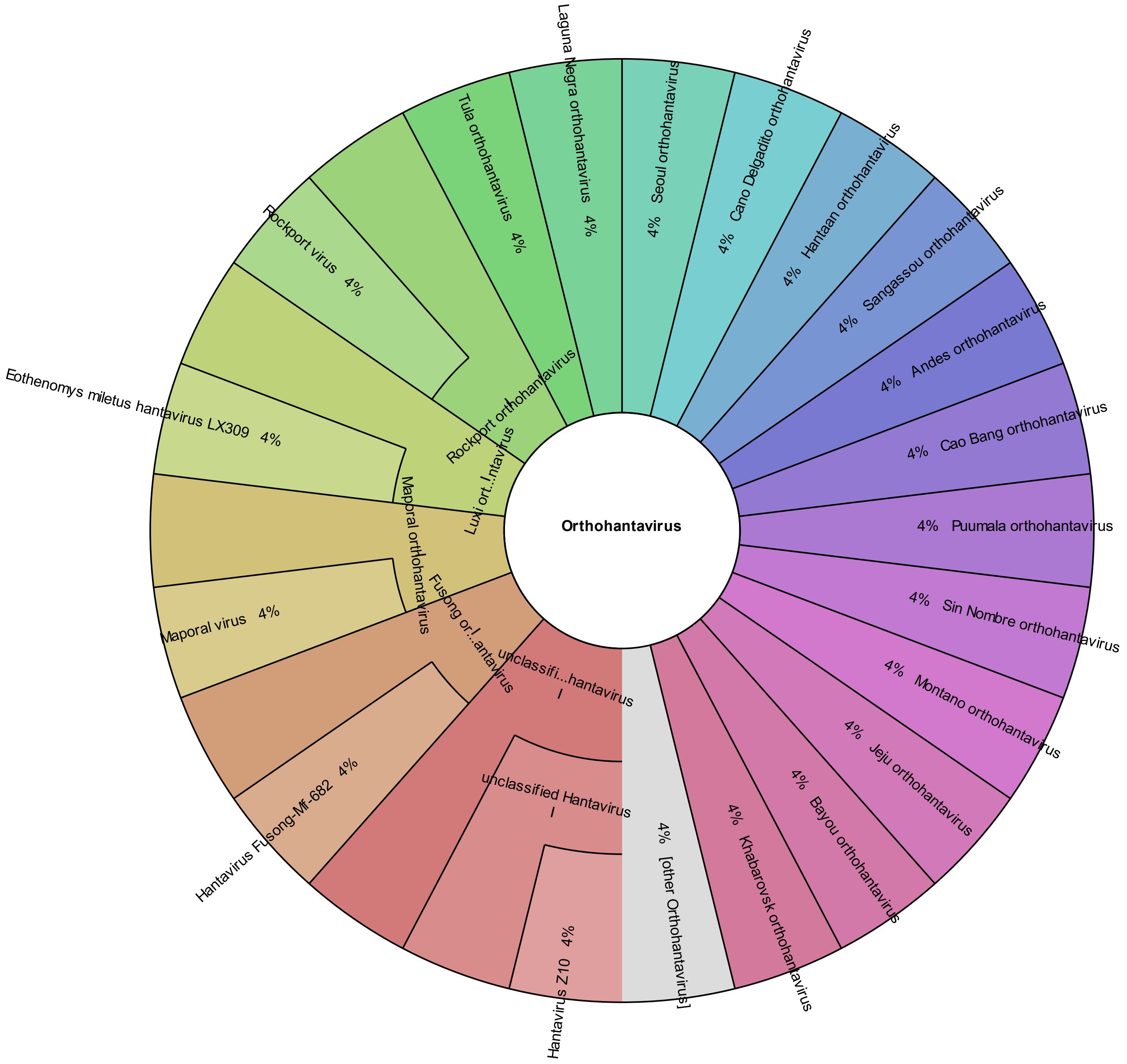

Supplemental Figure 1. Krona plot displaying multiple hits of orthohantaviruses for MSB:Mamm:332771. A snapshot of Krona plot is shown at the *Orthohantavirus* genus rank. Total slice represents an orthohantavirus that reads may associate with for potential hits. Unclassified hits shown account for 12% of potential hits, while Fǔsōng virus (FUSV), Maporal virus (MPAV), Lúxī virus (LUXV), and Rockport virus (RKPV) account for 8% of hits each. Total reads for *Orthohantavirus* is 38,325 of 14, 907, 288 reads generated. Virus names were generated by the Kraken2 software and may not be reflective of updated ICTV nomenclature.

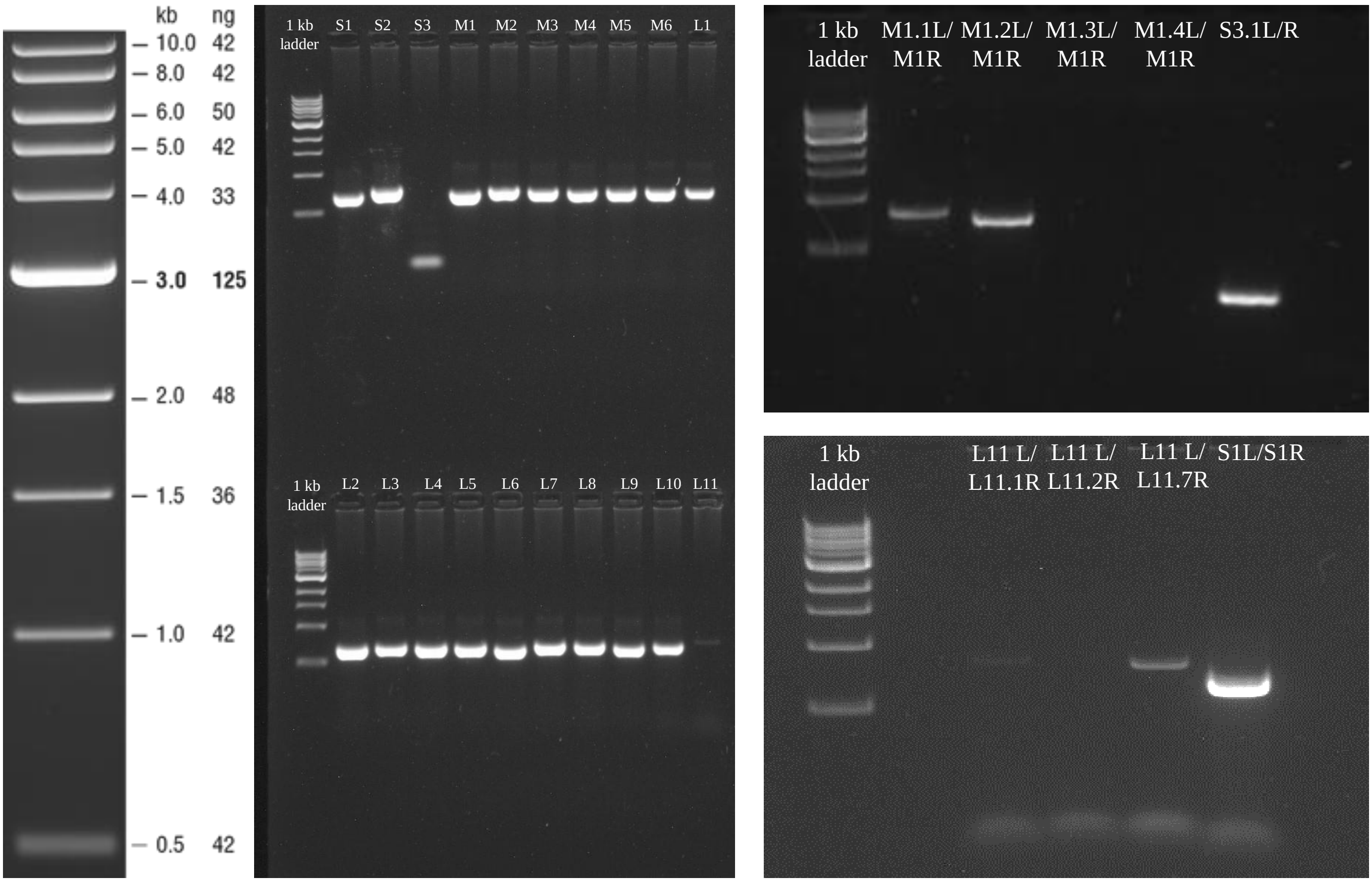

Supplemental Figure 2. Confirmation of product for LSCV amplification using PCR tiling primers. Gels displayed show bands for each respective primer name. These products were purified and Sanger-sequenced to confirm genome for "LSCV". Ladder (left) was used to show expected band length. Primer sets were used to determine amplification of "LSCV" (middle). Optimized PCR conditions and re-designed primers were necessary to amplify some S and L segment fragments (right).

S Segment

Virus	LSCV	MTNV	ELMCV	RSV	BCCV	BAYV	MULV	SNV	NYV	CADV	CHOV	ANDV	RIOMV	LANV	TULV	PUUV	HTNV	SEOV	DOBV
**LSCV**	-	29.9	30.9	31.7	35.7	36.4	36.6	35.9	36.3	36.8	33.5	34.3	35.4	35.7	38.7	38.9	44.0	43.3	44.0
**MTNV**	6.5	-	30.0	30.2	35.3	36.9	37.0	37.1	35.0	36.6	36.2	33.5	37.1	35.3	38.3	38.9	43.6	43.2	45.2
**ELMCV**	9.1	9.6	-	25.7	35.0	34.6	35.9	35.0	34.5	34.7	34.9	33.3	34.4	35.2	38.6	38.5	43.7	43.7	44.2
**RSV**	11.2	11.0	8.9	-	34.5	32.9	35.6	34.0	35.6	34.0	33.1	33.4	33.3	32.8	36.7	35.4	44.2	43.7	43.0
**BCCV**	16.1	16.1	16.6	18.0	-	25.4	26.5	35.2	34.5	34.8	33.0	32.1	32.0	31.3	39.3	39.1	42.1	42.5	43.2
**BAYV**	15.6	14.2	15.4	16.4	7.7	-	24.0	33.5	32.3	34.1	32.6	30.6	31.2	30.8	37.0	38.6	40.7	45.0	41.7
**MULV**	18.2	18.5	17.8	18.9	10.0	7.2	-	34.5	34.0	33.7	33.6	32.7	33.8	32.6	38.7	38.9	42.4	44.7	43.5
**SNV**	14.9	13.1	15.4	16.8	16.1	13.1	17.1	-	22.5	33.0	34.0	32.9	33.4	32.0	37.0	39.4	42.2	42.6	41.4
**NYV**	13.8	14.2	16.1	17.5	14.9	12.8	16.4	7.0	-	32.7	32.8	31.7	33.6	32.5	38.1	38.4	44.1	42.9	43.1
**CADV**	18.7	18.0	17.3	19.2	17.3	16.6	16.8	16.4	15.6	-	32.4	32.8	32.4	33.6	38.5	39.0	44.0	43.0	44.1
**CHOV**	13.8	13.5	16.1	17.3	14.5	11.4	15.4	12.1	11.7	14.7	-	29.8	29.8	29.1	39.4	39.6	42.7	43.5	44.1
**ANDV**	15.6	15.4	17.1	18.2	13.5	11.7	14.0	14.0	12.4	14.5	9.8	-	27.5	27.8	38.3	38.6	42.7	44.5	43.4
**RIOMV**	16.6	17.1	17.3	18.9	13.3	11.9	14.0	15.4	13.3	15.2	10.2	8.9	-	24.0	37.7	38.2	42.0	43.6	46.3
**LANV**	16.8	17.5	17.8	19.4	14.5	12.8	14.5	14.7	13.1	15.9	12.1	9.6	7.2	-	38.1	37.7	40.9	42.5	42.9
**TULV**	24.0	24.5	25.2	24.9	32.3	23.1	24.0	25.4	26.8	26.3	23.3	24.0	24.0	24.0	-	31.2	42.5	43.4	42.7
**PUUV**	28.0	29.0	29.4	28.7	26.2	26.9	26.2	29.2	29.2	28.7	29.2	27.8	27.1	27.6	20.3	-	43.3	44.5	43.2
**HTNV**	36.5	36.8	37.5	37.7	35.4	35.6	37.0	37.0	36.5	37.7	35.6	34.9	35.4	35.6	35.8	38.9	-	28.2	29.0
**SEOV**	36.0	36.5	38.2	38.4	36.1	36.5	37.7	37.5	36.5	37.2	36.8	35.1	36.1	36.1	36.5	37.1	16.8	-	29.1
**DOBV**	36.3	37.2	37.5	38.2	36.3	36.3	36.5	37.0	36.3	37.5	35.6	35.1	36.5	35.8	36.2	38.2	17.0	18.4	-

M Segment

Virus	LSCV	MTNV	ELMCV	BCCV	BAYV	MULV	SNV	NYV	CADV	CHOV	ANDV	RIOMV	LANV	TULV	PUUV	HTNV	SEOV	DOBV
**LSCV**	-	24.9	28.7	28.7	29.0	29.2	26.8	27.1	31.1	29.9	31.8	30.7	31.5	33.8	35.3	41.0	41.9	41.3
**MTNV**	11.8	-	30.0	29.8	30.1	29.0	28.7	28.3	31.8	31.9	31.5	31.2	32.1	35.5	36.0	41.1	42.7	42.5
**ELMCV**	20.4	30.3	-	29.7	30.3	29.8	29.4	29.7	30.8	31.3	32.6	31.3	31.6	34.3	36.2	42.0	42.2	42.0
**BCCV**	21.7	22.1	23.8	-	23.0	23.1	28.3	28.7	31.2	30.0	29.5	30.2	29.9	34.8	37.0	42.0	41.1	42.4
**BAYV**	21.9	21.4	23.4	11.5	-	23.6	28.8	28.7	31.2	29.9	29.7	29.9	31.0	35.4	36.4	41.8	41.8	42.5
**MULV**	22.1	21.2	24.7	12.3	11.3	-	29.7	29.7	30.5	29.1	29.8	30.0	30.7	34.7	36.2	41.7	41.1	42.0
**SNV**	17.5	17.5	20.4	20.0	19.2	20.7	-	19.2	29.7	29.7	29.5	29.1	29.8	33.9	35.2	43.0	43.5	42.6
**NYV**	17.4	17.7	20.9	20.7	20.5	20.5	4.3	-	30.2	29.8	29.3	29.7	29.3	34.1	35.5	41.9	42.9	42.9
**CADV**	25.0	25.3	26.3	25.1	24.2	25.5	22.8	23.7	-	30.4	30.5	31.2	30.7	35.3	37.2	42.1	41.8	42.0
**CHOV**	23.5	24.5	25.2	23.8	22.6	24.4	21.5	22.8	23.6	-	27.0	26.9	28.5	36.1	37.3	42.7	42.9	41.8
**ANDV**	25.7	25.9	26.7	24.6	24.2	24.8	22.1	22.7	25.9	17.1	-	24.7	25.8	35.5	35.9	42.4	42.6	42.5
**RIOMV**	24.9	25.7	25.5	24.7	24.2	25.2	22.5	23.4	25.3	16.9	12.3	-	22.6	35.8	36.7	42.5	43.1	42.5
**LANV**	25.5	27.1	25.7	24.2	24.4	26.2	23.2	23.5	26.1	18.4	13.5	8.8	-	35.4	35.2	42.1	43.5	43.0
**TULV**	31.5	32.2	31.5	31.7	31.9	32.0	30.8	30.6	34.0	33.6	32.1	31.8	32.6	-	28.7	41.5	41.6	42.1
**PUUV**	33.5	34.3	33.3	34.6	35.4	35.1	33.3	32.7	35.6	35.3	33.3	33.8	34.1	21.1	-	42.0	42.0	42.3
**HTNV**	42.3	43.4	43.5	35.3	44.7	45.1	45.1	45.2	44.6	45.6	45.0	44.6	45.3	44.6	46.5	-	28.8	29.4
**SEOV**	44.9	45.4	45.4	36.4	45.6	45.6	46.9	46.9	45.7	46.5	45.7	45.7	45.9	45.2	46.2	22.9	-	29.8
**DOBV**	44.2	44.8	45.2	36.5	45.5	45.5	46.2	46.0	45.6	45.3	45.7	45.2	45.8	44.9	44.7	21.6	22.8	-

L Segment

Virus	LSCV	MTNV	BAYV	SNV	NYV	CADV	ANDV	RIOMV	TULV	PUUV	HTNV	SEOV	DOBV
**LSCV**	-	22.1	25.8	24.9	24.4	24.9	25.8	25.0	28.6	28.5	34.6	33.1	33.9
**MTNV**	9.6	-	26.1	24.8	24.4	25.5	25.9	25.3	28.7	28.6	34.1	33.6	33.9
**BAYV**	14.9	16.2	-	25.4	25.0	24.1	24.9	25.1	28.4	28.4	34.2	33.4	33.8
**SNV**	11.6	13.1	14.8	-	19.7	25.0	24.7	25.2	28.6	29.1	34.4	32.6	33.4
**NYV**	12.0	13.5	14.3	4.9	-	24.7	24.9	24.9	29.0	28.5	33.7	34.1	33.7
**CADV**	14.9	15.3	12.9	13.3	13.5	-	24.5	24.6	28.5	27.9	34.4	33.4	34.3
**ANDV**	14.8	15.8	14.1	13.2	13.9	12.8	-	23.0	28.7	28.7	34.2	34.2	34.0
**RIOMV**	13.7	14.2	12.6	13.0	13.1	12.3	9.8	-	29.1	28.7	34.2	34.5	33.7
**TULV**	21.7	22.0	21.6	21.4	21.8	21.8	21.8	21.6	-	24.9	34.7	34.1	33.9
**PUUV**	22.9	22.8	22.3	21.9	22.0	22.1	22.6	22.3	14.8	-	34.2	33.4	33.5
**HTNV**	30.9	31.2	30.5	30.7	30.8	30.8	31.3	30.4	31.4	30.9	-	25.7	25.3
**SEOV**	30.8	30.9	30.7	30.8	30.8	30.8	31.9	30.8	31.1	31.3	15.0	-	25.0
**DOBV**	30.5	30.5	30.7	30.4	30.2	30.5	31.7	30.9	31.0	30.4	14.8	14.5	-

Supplemental Figure 3. Matrix identity for S, M and L Segment against MSB:Mamm:332771 (LSCV). Percent identity matrix was generated using Clustal Omega software. Top (white) indicates percentage differences in nucleotides. Bottom (gray) indicates percentage differences in amino acids. Sequences used were complete segments and translated reading regions with amino acids only. Abbreviations are described in the manuscript figures and Supplemental Table 4.

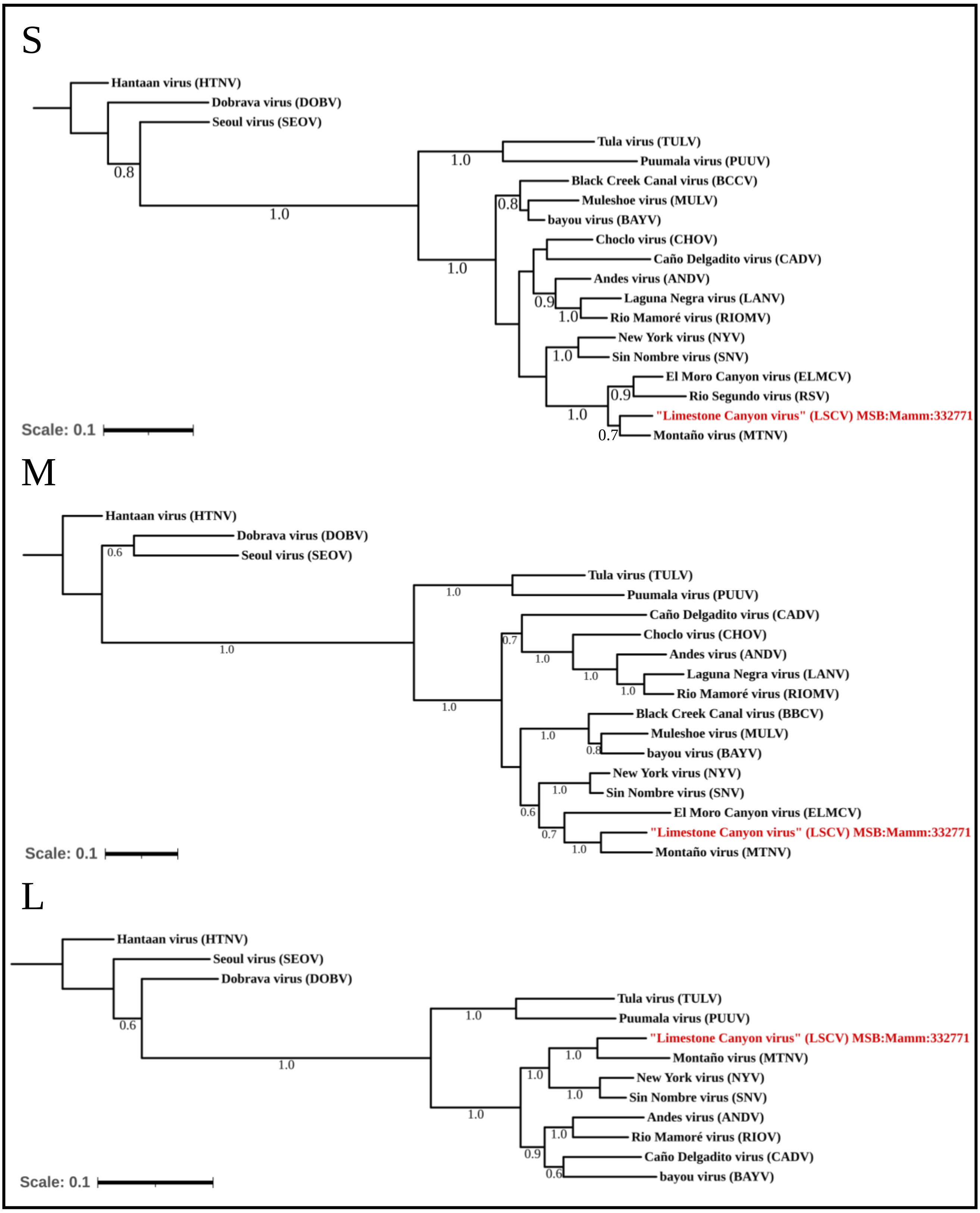

Supplemental Figure 4. Phylogeny of amino acids of current reference genomes for each segment. Reference genomes obtained through NCBI for S, M, and L were used for translated regions against MSB:Mamm:332771. Bootstrap values are indicated.

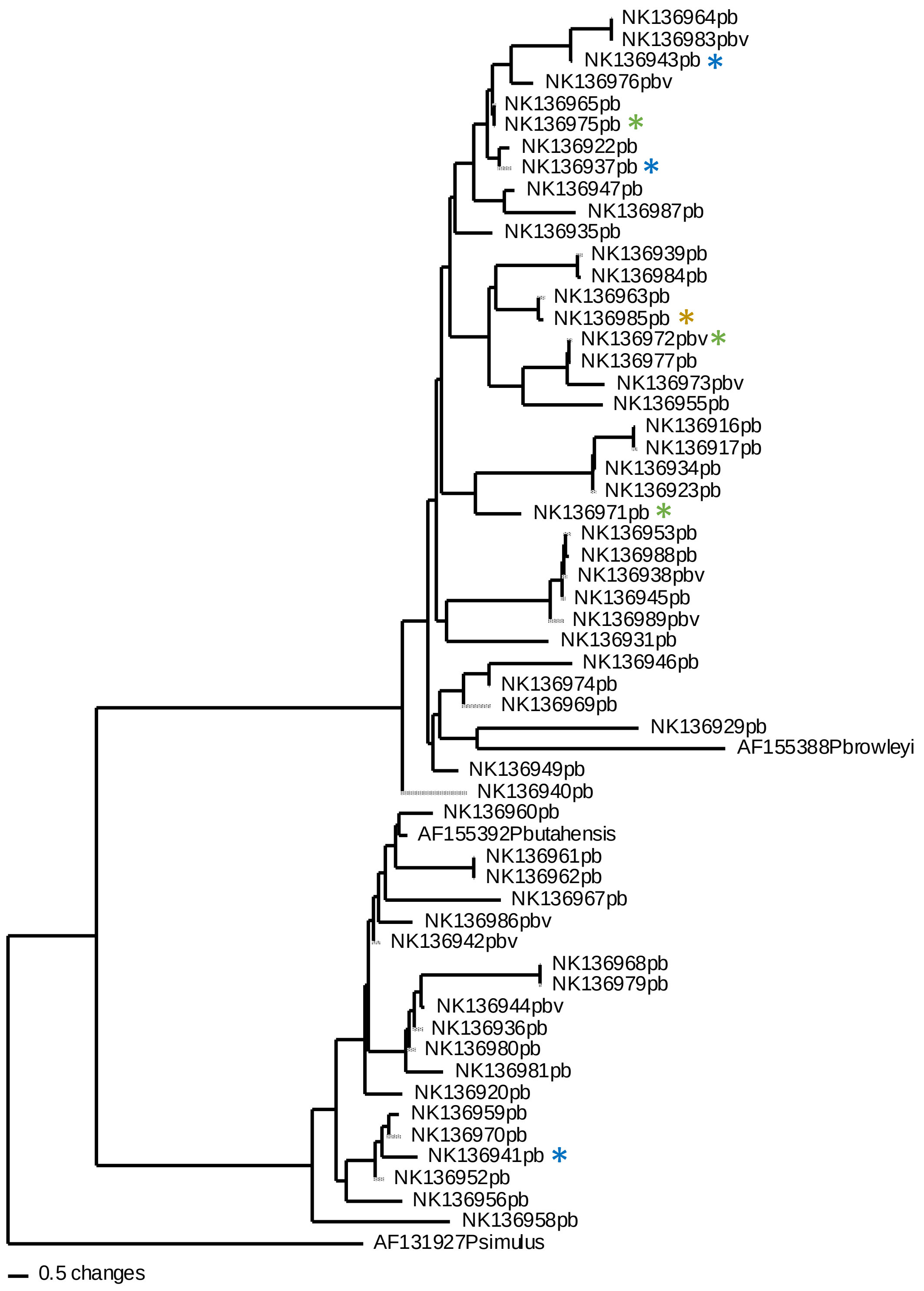

Supplemental Figure 5. Tree obtained from a Neighbor-Joining analysis of *Peromyscus boylii* sequences found in 2006 compared with DNA sequences (AF numbers) from Tiemann-Boege et al. (*64*). The mice fom this study (identified by NK numbers) were found in two clades. A *P. stimulus* sequence was used as an outgroup. * indicate associate sites: Site 1 (blue), Site 2 (green), and Site 3 (gold).

Contigs (Node)	Segment	Coding Gene	Nucleotides	BLAST result	% Similarity
Contig 1 (Node 2)	Small (S)	Nucleocapsid	1,933	"LSCV" nucleocapsid	94.29
Contig 2 (Node 11)	Small (S)	Nucleocapsid	245	"LSCV" nucleocapsid	93.88
Contig 3 (Node 13)	Small (S)	Nucleocapsid	242	"LSCV" nucleocapsid	91.29
Contig 4 (Node 18)	Small (S)	Nucleocapsid	225	"LSCV" nucleocapsid	98.31
Contig 5 (Node 3)	Medium (M)	Glycoproteins	1,352	"LSCV" glycoproteins	93.03
Contig 6 (Node 1)	Large (L)	Large protein	5,373	MTNV large protein	79.31
Contig 7 (Node 4)	Large (L)	Large protein	781	SNV (BV121) large protein	76.18
Contig 8 (Node 9)	Large (L)	Large protein	274	ANDV (ARLA17/NRC2) large protein	82.27

Supplemental Table 1. Contig sequences generated by *de novo* assembly for MSB332771 using whole-genome sequencing. Eight of 18 nodes are represented, with those not shown not being orthohantavirus hits or yielding "no result". ANDV: Andes virus. "LSCV": "Limestone Canyon virus", MTNV: Montaño virus, SNV: Sin Nombre virus. BLAST: Basical Local Alignment Search Tool.

		Coverage				
**Number**	**Primer Sequence (5' -> 3')**	**Start**	**Stop**	**Name**	**Sense Strand**	**Pool**
**1**	TACTACTGCAGAAGCTGGCATG	31	53	LSCV_S_Segment_1_LEFT	+	1
**2**	CGGTCTGTCCAATCCTTCACAA	754	732	LSCV_S_Segment_1_RIGHT	-	1
**3**	ACACCAGGACGTTTTCGAACAA	629	651	LSCV_S_Segment_2_LEFT	+	2
**4**	CACATGTCTAAATACAATGC	1764	1744	LSCV_S_Segment_3.8_Right	-	2
**5**	TAGACTCCGCACGAAGAAGC	1	20	LSCV_M_Segment_1.2_LEFT	+	1
**6**	TGCTGATCTATAATCTTCCATCATTGGT	823	795	LSCV_M_Segment_1_RIGHT	-	1
**7**	TGCTGTCGAATTGGAAAAGCTAAT	690	714	LSCV_M_Segment_2_LEFT	+	2
**8**	GCCACCCCAGGGATTAAAGAAA	1422	1400	LSCV_M_Segment_2_RIGHT	-	2
**9**	TCATCTGCCAACGAGTTGATCA	1286	1308	LSCV_M_Segment_3_LEFT	+	1
**10**	GCTGTGTCAGACCAACCTGTTT	2004	1982	LSCV_M_Segment_3_RIGHT	-	1
**11**	TGTTATGTTGGTCTAGTCTGGTGT	1900	1924	LSCV_M_Segment_4_LEFT	+	2
**12**	GCTTGAGGATTAGTCCACCTTGT	2603	2580	LSCV_M_Segment_4_RIGHT	-	2
**13**	TCTTGTCACATCATCAGTGAAGGT	2490	2514	LSCV_M_Segment_5_LEFT	+	1
**14**	TGCTGATGCAAGAAGTCCTTCC	3199	1377	LSCV_M_Segment_5_RIGHT	-	1
**15**	ACCTAGTACTTAACAGAGATGTTTCATTT	2876	2905	LSCV_M_Segment_6_LEFT	+	2
**16**	GCCCAGTTATAAGCAAATATGATTATATGGT	3590	3559	LSCV_M_Segment_6_RIGHT	-	2
**17**	TCGATAAATACACCAGAGAGTCAAAGA	27	54	LSCV_L_Segment_1_LEFT	+	1
**18**	AGGCTCTAAAAGAGGTTGAGGTT	730	707	LSCV_L_Segment_1_RIGHT	-	1
**19**	AGAAAGATCTTCATTGGAGGCTATGT	621	647	LSCV_L_Segment_2_LEFT	+	2
**20**	ATTGCCGTTGTAGTTTCCCCAA	1332	1310	LSCV_L_Segment_2_RIGHT	-	2
**21**	TGTTTGGGGAAGTAATAGAGAGCA	1208	1232	LSCV_L_Segment_3_LEFT	+	1
**22**	ACCAGAATATAAAGATGTCACAGAAGGT	1936	1908	LSCV_L_Segment_3_RIGHT	-	1
**23**	CTTTACAACATGCCTTGCGGTC	1805	1827	LSCV_L_Segment_4_LEFT	+	2
**24**	AGCTCCTGACATACCTTTCAAACT	2527	2503	LSCV_L_Segment_4_RIGHT	-	2
**25**	ACTTACAGGCTAAGTCACAAGTGT	2417	2441	LSCV_L_Segment_5_LEFT	+	1
**26**	ACAGCTGATGAATGTTCCTCCA	3129	3107	LSCV_L_Segment_5_RIGHT	-	1
**27**	GTTGTGTTGTTGATGCATTACGGA	3026	3050	LSCV_L_Segment_6_LEFT	+	2
**28**	ATGGCAAGTTGAGCAAGTTGTG	3705	3683	LSCV_L_Segment_6_RIGHT	-	2
**29**	GGTTCATTATCAGATCTACCTGGGT	3589	3614	LSCV_L_Segment_7_LEFT	+	1
**30**	TGCAAGGACCTCACGAAATGTT	4312	4290	LSCV_L_Segment_7_RIGHT	-	1
**31**	CCTAGCATTGTCACTGCCATGA	4186	4208	LSCV_L_Segment_8_LEFT	+	2
**32**	TCCGTGTTGCAGCTAAATCTTCT	4910	4887	LSCV_L_Segment_8_RIGHT	-	2
**33**	ACTATCAAGGTAAGACCGAAAAAGGA	4810	4836	LSCV_L_Segment_9_LEFT	+	1
**34**	GGTTGTTTAGTTCTAAGACCTGTACG	5499	5473	LSCV_L_Segment_9_RIGHT	-	1
**35**	GCCCGTAGCCTTGTTCTGTTTA	5383	5405	LSCV_L_Segment_10_LEFT	+	2
**36**	TTAATGTCAGTGTCCTCTGTCTGT	6093	6069	LSCV_L_Segment_10_RIGHT	-	2
**37**	TCCTAAATCCCATAATTGC	5678	5697	LSCV_L_Segment_11_LEFT	+	1
**38**	AGTAGTATGCTCCGAGAAAAGAG	6537	6514	LSCV_L_Segment_11.7_RIGHT	-	1

Supplemental Table 2. PCR tiling primers used to Sanger-sequence "Limestone Canyon virus (LSCV)" genomes. Primers were generated using PRIMAL SCHEME software.

MSB Number	NK Number	Species	Latitude	Longitude	County	Locality
**MSB:Mamm:332771**	**-**	***P. boylii***	**34.3721N**	**106.2454W**	**Torrance**	**Red Canyon**
MSB:Mamm:264851	NK 136901	*P. eremicus*	32.09599N	108.97421W	Hidalgo	Granite Gap
MSB:Mamm:264852	NK 136902	*P. eremicus*	32.09599N	108.97421W	Hidalgo	Granite Gap
MSB:Mamm:264853	NK 136903	*P. eremicus*	32.09599N	108.97421W	Hidalgo	Granite Gap
MSB:Mamm:264854	NK 136904	*P. eremicus*	32.09599N	108.97421W	Hidalgo	Granite Gap
MSB:Mamm:264855	NK 136905	*P. eremicus*	32.09599N	108.97421W	Hidalgo	Granite Gap
MSB:Mamm:264856	NK 136906	*P. eremicus*	32.09599N	108.97421W	Hidalgo	Granite Gap
MSB:Mamm:264857	NK 136907	*P. eremicus*	32.09599N	108.97421W	Hidalgo	Granite Gap
MSB:Mamm:264858	NK 136908	*P. eremicus*	32.09599N	108.97421W	Hidalgo	Granite Gap
MSB:Mamm:264859	NK 136909	*P. eremicus*	32.09599N	108.97421W	Hidalgo	Granite Gap
MSB:Mamm:264860	NK 136910	*P. eremicus*	32.09599N	108.97421W	Hidalgo	Granite Gap
MSB:Mamm:264861	NK 136911	*P. eremicus*	32.09599N	108.97421W	Hidalgo	Granite Gap
MSB:Mamm:264862	NK 136912	*P. eremicus*	32.09599N	108.97421W	Hidalgo	Granite Gap
MSB:Mamm:264863	NK 136913	*P. eremicus*	32.09599N	108.97421W	Hidalgo	Granite Gap
MSB:Mamm:264864	NK 136914	*P. eremicus*	32.09599N	108.97421W	Hidalgo	Granite Gap
MSB:Mamm:264865	NK 136915	*P. maniculatus*	32.64148N	108.57881W	Grant	Edgar Place Road
MSB:Mamm:264866	NK 136916	*P. boylii*	32.64148N	108.57881W	Grant	Edgar Place Road
MSB:Mamm:264867	NK 136917	*P. boylii*	32.64148N	108.57881W	Grant	Edgar Place Road
MSB:Mamm:264868	NK 136919	*P. truei*	32.53550N	108.46131W	Grant	Gold Gulch Road
MSB:Mamm:264869	NK 136919	*P. boylii*	32.53550N	108.46131W	Grant	Gold Gulch Road
MSB:Mamm:264870	NK 136920	*P. truei*	32.53550N	108.46131W	Grant	Gold Gulch Road
MSB:Mamm:264871	NK 136921	*P. boylii*	32.53550N	108.46131W	Grant	Gold Gulch Road
MSB:Mamm:264872	NK 136922	*P. boylii*	32.53550N	108.46131W	Grant	Gold Gulch Road
MSB:Mamm:264873	NK 136923	*P. leucopus*	32.53550N	108.46131W	Grant	Gold Gulch Road
MSB:Mamm:264874	NK 136924	*P. truei*	32.53550N	108.46131W	Grant	Gold Gulch Road
MSB:Mamm:264875	NK 136925	*P. truei*	32.53550N	108.46131W	Grant	Gold Gulch Road
MSB:Mamm:264876	NK 136926	*P. truei*	32.53550N	108.46131W	Grant	Gold Gulch Road
MSB:Mamm:264877	NK 136927	*P. truei*	32.53550N	108.46131W	Grant	Gold Gulch Road
MSB:Mamm:264878	NK 136928	*P. boylii*	32.53550N	108.46131W	Grant	Gold Gulch Road
MSB:Mamm:264879	NK 136929	*P. truei*	32.53550N	108.46131W	Grant	Gold Gulch Road
MSB:Mamm:264880	NK 136930	*P. boylii*	32.53550N	108.46131W	Grant	Gold Gulch Road
MSB:Mamm:264881	NK 136931	*P. truei*	32.53550N	108.46131W	Grant	Gold Gulch Road
MSB:Mamm:264882	NK 136932	*P. truei*	32.53550N	108.46131W	Grant	Gold Gulch Road
MSB:Mamm:264883	NK 136933	*P. boylii*	32.53550N	108.46131W	Grant	Gold Gulch Road
MSB:Mamm:264884	NK 136934	*P. boylii*	32.54056N	108.47015W	Grant	Gold Gulch Road
MSB:Mamm:264885	NK 136935	*P. boylii*	32.54056N	108.47015W	Grant	Gold Gulch Road
MSB:Mamm:264886	NK 136936	*P. boylii*	32.54056N	108.47015W	Grant	Gold Gulch Road
MSB:Mamm:264887	NK 136937	*P. boylii*	32.61938N	108.48524W	Grant	Tyrone Thompson Road
MSB:Mamm:264888	NK 136938	*P. boylii*	32.61938N	108.48524W	Grant	Tyrone Thompson Road
MSB:Mamm:264889	NK 136939	*P. boylii*	32.61938N	108.48524W	Grant	Tyrone Thompson Road
MSB:Mamm:264890	NK 136940	*P. boylii*	32.61938N	108.48524W	Grant	Tyrone Thompson Road
MSB:Mamm:264891	NK 136941	*P. boylii*	32.61938N	108.48524W	Grant	Tyrone Thompson Road
MSB:Mamm:264892	NK 136942	*P. boylii*	32.61938N	108.48524W	Grant	Tyrone Thompson Road
MSB:Mamm:264893	NK 136943	*P. boylii*	32.61938N	108.48524W	Grant	Tyrone Thompson Road
MSB:Mamm:264894	NK 136944	*P. boylii*	32.61938N	108.48524W	Grant	Tyrone Thompson Road
MSB:Mamm:264897	NK 136945	*P. boylii*	32.61938N	108.48524W	Grant	Tyrone Thompson Road
MSB:Mamm:264898	NK 136946	*P. boylii*	32.61938N	108.48524W	Grant	Tyrone Thompson Road
MSB:Mamm:264899	NK 136947	*P. truei*	32.61938N	108.48524W	Grant	Tyrone Thompson Road
MSB:Mamm:264900	NK 136948	*P. boylii*	32.61283N	108.48899W	Grant	Tyrone Thompson Road
MSB:Mamm:264901	NK 136949	*P. boylii*	32.61283N	108.48899W	Grant	Tyrone Thompson Road
MSB:Mamm:264902	NK 136950	*P. truei*	32.61283N	108.48899W	Grant	Tyrone Thompson Road
MSB:Mamm:264903	NK 136951	*P. boylii*	32.61283N	108.48899W	Grant	Tyrone Thompson Road
MSB:Mamm:264904	NK 136952	*P. boylii*	32.61283N	108.48899W	Grant	Tyrone Thompson Road
MSB:Mamm:264905	NK 136953	*P. truei*	32.61283N	108.48899W	Grant	Tyrone Thompson Road

MSB:Mamm:264906	NK 136954	*P. boylii*	32.61283N	108.48899W	Grant	Tyrone Thompson Road
MSB:Mamm:264907	NK 136955	*P. boylii*	32.61283N	108.48899W	Grant	Tyrone Thompson Road
MSB:Mamm:264908	NK 136956	*P. truei*	32.61283N	108.48899W	Grant	Tyrone Thompson Road
MSB:Mamm:264909	NK 136957	*P. boylii*	32.61283N	108.48899W	Grant	Tyrone Thompson Road
MSB:Mamm:264910	NK 136958	*P. boylii*	32.61283N	108.48899W	Grant	Tyrone Thompson Road
MSB:Mamm:264911	NK 136959	*P. boylii*	32.61283N	108.48899W	Grant	Tyrone Thompson Road
MSB:Mamm:264912	NK 136960	*P. boylii*	32.61283N	108.48899W	Grant	Tyrone Thompson Road
MSB:Mamm:264913	NK 136961	*P. boylii*	32.61283N	108.48899W	Grant	Tyrone Thompson Road
MSB:Mamm:264914	NK 136962	*P. boylii*	32.61283N	108.48899W	Grant	Tyrone Thompson Road
MSB:Mamm:264915	NK 136963	*P. boylii*	32.61283N	108.48899W	Grant	Tyrone Thompson Road
MSB:Mamm:264916	NK 136964	*P. boylii*	32.61283N	108.48899W	Grant	Tyrone Thompson Road
MSB:Mamm:264917	NK 136965	*P. truei*	32.61283N	108.48899W	Grant	Tyrone Thompson Road
MSB:Mamm:264918	NK 136966	*P. boylii*	32.61283N	108.48899W	Grant	Tyrone Thompson Road
MSB:Mamm:264919	NK 136967	*P. boylii*	32.61283N	108.48899W	Grant	Tyrone Thompson Road
MSB:Mamm:264920	NK 136968	*P. boylii*	32.61283N	108.48899W	Grant	Tyrone Thompson Road
MSB:Mamm:264921	NK 136969	*P. boylii*	32.61283N	108.48899W	Grant	Tyrone Thompson Road
MSB:Mamm:264922	NK 136970	*P. boylii*	32.61283N	108.48899W	Grant	Tyrone Thompson Road
MSB:Mamm:264923	NK 136971	*P. boylii*	32.61283N	108.48899W	Grant	Tyrone Thompson Road
MSB:Mamm:264924	NK 136972	*P. boylii*	32.61283N	108.48899W	Grant	Tyrone Thompson Road
MSB:Mamm:264925	NK 136973	*P. boylii*	32.61283N	108.48899W	Grant	Tyrone Thompson Road
MSB:Mamm:264926	NK 136974	*P. boylii*	32.61283N	108.48899W	Grant	Tyrone Thompson Road
MSB:Mamm:264927	NK 136975	*P. boylii*	32.61283N	108.48899W	Grant	Tyrone Thompson Road
MSB:Mamm:264928	NK 136976	*P. boylii*	32.61283N	108.48899W	Grant	Tyrone Thompson Road
MSB:Mamm:264929	NK 136977	*P. truei*	32.82832N	108.35786W	Grant	Bear Mountain Road
MSB:Mamm:264930	NK 136978	*P. boylii*	32.82832N	108.35786W	Grant	Bear Mountain Road
MSB:Mamm:264931	NK 136979	*P. boylii*	32.82832N	108.35786W	Grant	Bear Mountain Road
MSB:Mamm:264932	NK 136980	*P. boylii*	32.82832N	108.35786W	Grant	Bear Mountain Road
MSB:Mamm:264933	NK 136981	*P. boylii*	32.61283N	108.48899W	Grant	Tyrone Thompson Road
MSB:Mamm:264934	NK 136982	*P. boylii*	32.61283N	108.48899W	Grant	Tyrone Thompson Road
MSB:Mamm:264935	NK 136983	*P. boylii*	32.62787N	108.47670W	Grant	Forest Road 136
MSB:Mamm:264936	NK 136984	*P. boylii*	32.62787N	108.47670W	Grant	Forest Road 136
MSB:Mamm:264937	NK 136985	*P. boylii*	32.62284N	108.42790W	Grant	Silby Road
MSB:Mamm:264938	NK 136986	*P. boylii*	32.62284N	108.42790W	Grant	Silby Road
MSB:Mamm:264939	NK 136987	*P. boylii*	32.62284N	108.42790W	Grant	Silby Road
MSB:Mamm:264940	NK 136988	*P. boylii*	32.62284N	108.42790W	Grant	Silby Road

Supplemental Table 3. Specimens examined. The Torrance County rodent (2019) captured in Red Canyon, Cibola National Forest is listed at the top (gray). Hidalgo and Grant County specimens (2006) were collected in the Burro Mountains, Gila National Forest and are listed subsequently.

Virus name	S segment	M Segment	L Segment
**Hantaan virus (HTNV)**	*NC_005218*	*NC_005219*	X55901.1
**Seoul virus (SEOV)**	AY273791.1	S47716.1	X56492.1
**Puumala virus (PUUV)**	X61035.1	X61034.1	Z66548.1
**Dobrava-Belgrade virus (DOBV)**	AJ410615.1	AJ410616.1	AJ410617.1
**Tula virus (TULV)**	Z49915.1	Z69993.1	AJ006537.1
**Sin Nombre virus (SNV)**	L25784.1	L25783.1	L37901.1
**El Moro Canyon virus (ELMCV)**	*NC_038423.1*	*NC_038424.1*	-
**Monongahela virus (MNGV)**	MH539867.1	MH539866.1	MH539865.1
**New York virus (NYV)**	MG717391.1	MG717392.1	MG717393.1
**Muleshoe virus (MULV)**	KX066124.1	KX066125.1	-
**Black Creek Canal virus (BCCV)**	L39949.1	L39950.1	L39951.1
**Bayou virus (BAYV)**	L36929.1	L36930.1	GQ244526.1
**Rio Segundo virus (RSV)**	U18100.1	-	-
**Cano Delgadito virus (CADV)**	*NC_034528.1*	*NC_034525.1*	GQ200821.1
**Choclo virus (CHOV)**	DQ285046.1	DQ285047.1	-
**Andes virus (ANDV)**	AF291702.1	AF291703.2	AF291704.5
**Laguna Negra virus (LNV)**	AF005727.1	AF005728.1	-
**Rio Mamore virus (RIOMV)**	FJ532244.1	FJ608550.1	FJ809772.1
**Montano virus (MTNV)**	AB620100.1	AB620101.1	AB620102.1
**"Limestone Canyon virus" (LSCV)**	AF307322*	AF307323	-
**MSB:Mamm:332771**	OR148902	OR148903	OR148904
**MSB:Mamm:264924**	OR552611	OR552612	OR552613
**MSB:Mamm:264934**	OR552601	OR552602	OR552603
**MSB:Mamm:264927**	OR552607	OR552608	OR552609
**MSB:Mamm:264923**	OR552604	OR552605	OR552606
**MSB:Mamm:264891**	OR552620	OR552621	OR552622
**MSB:Mamm:264887**	OR552617	OR552618	OR552619
**MSB:Mamm:264937**	OR552614	OR552615	OR552616
**MSB:Mamm:264893**	OR552623	OR552624	OR552625

Supplemental Table 4. Orthohantavirus reference sequences through NCBI GenBank available for each segment with GenBank or RefSeq numbers used in phylogenetic analysis. *indicates partial sequence available and *italics* are only available in RefSeq numbers.
